# Characterization and Integration of Terahertz Technology within Microfluidic Platforms

**DOI:** 10.3390/mi9090453

**Published:** 2018-09-11

**Authors:** Salman Alfihed, Mark H. Bergen, Antonia Ciocoiu, Jonathan F. Holzman, Ian G. Foulds

**Affiliations:** 1 School of Engineering, University of British Columbia (UBC), Kelowna, BC V1V 1V7, Canada; mark.bergen@alumni.ubc.ca (M.H.B.); antonia.ciocoiu@alumni.ubc.ca (A.C.); Jonathan.Holzman@ubc.ca (J.F.H.); ian.foulds@ubc.ca (I.G.F.); 2 Materials Science Research Institute, King Abdulaziz City for Science and Technology (KACST), Riyadh 11442, Saudi Arabia

**Keywords:** THz time-domain spectroscopy, lab-on-a-chip, microfluidics, polymer absorption

## Abstract

In this work, the prospects of integrating terahertz (THz) time-domain spectroscopy (TDS) within polymer-based microfluidic platforms are investigated. The work considers platforms based upon the polar polymers polyethylene terephthalate (PET), polycarbonate (PC), polymethyl-methacrylate (PMMA), polydimethylsiloxane (PDMS), and the nonpolar polymers fluorinated ethylene propylene (FEP), polystyrene (PS), high-density polyethylene (HDPE), and ultra-high-molecular-weight polyethylene (UHMWPE). The THz absorption coefficients for these polymers are measured. Two microfluidic platforms are then designed, fabricated, and tested, with one being based upon PET, as a representative high-loss polar polymer, and one being based upon UHMWPE, as a representative low-loss nonpolar polymer. It is shown that the UHMWPE microfluidic platform yields reliable measurements of THz absorption coefficients up to a frequency of 1.75 THz, in contrast to the PET microfluidic platform, which functions only up to 1.38 THz. The distinction seen here is attributed to the differing levels of THz absorption and the manifestation of differing f for the systems. Such findings can play an important role in the future integration of THz technology and polymer-based microfluidic systems.

## 1. Introduction

Terahertz (THz) radiation is of growing interest in scientific applications [1,2]. Its frequencies lie within the electromagnetic spectrum between the microwave and infrared regions, from 0.1 to 10 THz. This makes its radiation both non-ionizing [1,2,3,4] and sensitive to many of the key rotational and vibrational modes that characterize biological samples [1,5]. The signatures of these bio-molecular absorption characteristics are typically gleaned via THz time-domain spectroscopy (THz-TDS) [6,7,8] in support of DNA analyses [8,9], cancerous cell detection [8,10], diabetes diagnostics [11], and other applications. The sensitivity that is achieved by such systems can be improved by scaling down their physical size, and for this reason there is growing interest in the integration of THz technology and microfluidic platforms [12].

Microfluidic platforms can enable highly sensitive characterizations of samples and reagents—but their integration with THz technology must carefully consider the incorporated materials. The materials must meet practical constraints, such as cost, chemical resistance, and bio-compatibility, without sacrificing the THz-TDS performance, as defined by its signal-to-noise ratio (SNR) and dynamic range [13,14,15]. In light of the practical constraints, microfluidic platforms have been developed in recent years with a wide variety of polymers [16], including polyethylene terephthalate (PET), polycarbonate (PC), polymethyl-methacrylate (PMMA), polydimethylsiloxane (PDMS), fluorinated ethylene propylene (FEP), polystyrene (PS), high-density polyethylene (HDPE), and ultra-high-molecular-weight polyethylene (UHMWPE) [17,18,19,20]. Such polymers can be patterned into microfluidic platforms at relatively low cost and can offer varying levels of chemical resistance and biocompatibility. Some, such as PMMA, offer strong chemical resistance [21,22,23], while others, such as PDMS, offer excellent biocompatibility [19]. Unfortunately, less is known about the THz absorption characteristics of the polymers and their impact in microfluidics-based THz-TDS systems. The proposed work seeks such knowledge.

In this work, the polar polymers PET, PC, PMMA, and PDMS and the nonpolar polymers FEP, PS, HDPE, and UHMWPE are characterized in terms of their THz absorption. The findings are used to test the prospects of THz-TDS analyses in polymer-based microfluidic platforms. The systems are contrasted in terms of their dynamic range and THz bandwidth. The findings and conclusions can support the development of future microfluidics-based THz-TDS systems.

## 2. Materials and Methods

### 2.1. Sample Preparation

The THz absorption characteristics being considered within this investigation are for samples of polar and nonpolar polymers. The selected polar polymers are PET, PC, PMMA, and PDMS, and the selected nonpolar polymers are FEP, PS, HDPE, and UHMWPE. The samples have thicknesses that are defined in consideration to their THz absorption coefficients and dynamic range. Specifically, each sample thickness is made equal to (roughly) twice the reciprocal of the average absorption coefficient across the frequencies spanning 0.5 to 2.0 THz. This optimal thickness creates a balance between the response of thin samples, which support high THz bandwidths but suffer from small THz amplitudes, and thick samples, which support large THz amplitudes but suffer from low THz bandwidths [24]. The frequency-dependent THz absorption coefficients were measured for all the polymer samples, and the samples were prepared with thicknesses being roughly equal to their optimal thicknesses. Three of the polymers, PC, PMMA, and HDPE, were available as bulk material at their optimal sample thicknesses. Four of the polymers, PET, FEP, PS, and UHMWPE, were fabricated into samples at the optimal thicknesses via thermal bonding of thin layered material. These latter samples were thermally bonded at 110 °C for 60 min, 265 °C for 60 min, 95 °C for 45 min, and 145 °C for 120 min, respectively. Thermal bonding mitigates the effects of internal reflections and resonance between layers during the THz-TDS measurements—which was found to be an issue for stacked polymer structures with air gaps between the layers. The remaining polymer, PDMS, was prepared by mixing its prepolymer base (SYLGARD^®^ 184 Silicone elastomer, Dow Chemical Company, Midland, MI, USA) and curing agent (SYLGARD^®^ 184 Silicone elastomer, Dow Chemical Company, Midland, MI, USA) at a 10% mass fraction of curing agent to prepolymer base. The 5 g PDMS mixture was then evacuated in a vacuum chamber for 30 min and cured within a 75 mm diameter Petri dish for 48 h at room temperature. Our prior studies have found that the THz absorption of PDMS increases as the curing temperature increases [25], and so this sample was cured over a long time at a low temperature. The measured thicknesses of the polymer samples, as measured at each centre, are shown in Table 1.

### 2.2. Microfluidic Platform Fabrication

**Figure 1:**
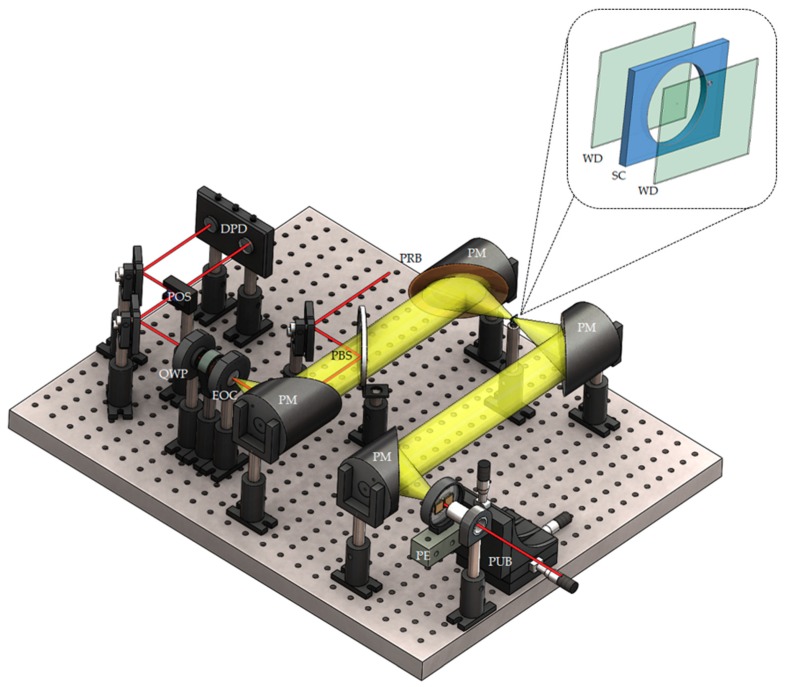
Isometric schematic of THz time-domain spectroscopy (THz-TDS) system, with the microfluidic platform at the top right. Ultrafast laser pulses are split into two beams: a pump beam (PUB), focused on the photoconductive SI-GaAs THz emitter (PE), and a probe beam (PRB). The THz beam (yellow) is generated at the emitter and then collected, collimated, and focused by parabolic mirrors (PMs). The probe beam is overlaid with the THz beam by a pellicle beamsplitter (PBS). An electro-optic ZnTe crystal (EOC) and quarter waveplate (QWP), allow the THz beam to modulate the probe beam’s polarization. A polarizing beamsplitter (POS) splits the probe beam into beams with orthogonal polarizations, and the power difference between the beams is measured by a differential photodetector (DPD). The microfluidic platform windows (WD) and PMMA spacer (SC) are shown in the inset.

The effects of polymer absorption on the functionality of THz-TDS analyses with microfluidics are demonstrated in this work for platforms incorporating two greatly contrasting polymers. The first polymer, PET, is selected because it is highly absorptive across the THz spectrum, while the second polymer, UHMWPE, is selected because it has low absorption across the THz spectrum. The microfluidic platforms are fabricated with 76 µm thick upper and lower windows, being comprised of PET or UHMWPE, between which lies a 2.4 mm thick and 50.8 mm diameter test cell. The test cell is surrounded by a PMMA layer that acts as a spacer between the windows and a perimeter that contains the test fluid. The structures were patterned with a CO_2_ laser cutter (H-Series 20 × 12 30 W, Full Spectrum Laser LLC, Las Vegas, NV, USA) and then sandwiched together via thermal bonding for the PET-PMMA interface, at a temperature of 105 °C for 30 min, or solvent bonding for the UHMWPE-PMMA interface, with methylene diphenyl disocyanate. A schematic of the fabricated microfluidic platform is shown in the inset of Figure 1.

### 2.3. Terahertz Time-Domain Spectroscopy

The THz-TDS system that measures the THz absorption coefficients of the test fluid within the microfluidic platform and the various polymers is shown as a schematic in Figure 1. For this system, a train of pulses with a duration of 100 fs, wavelength of 775 nm, and repetition rate of 90 MHz is generated by an ultrafast pulsed laser (FFS-SYS-2B, TOPTICA Photonics AG, Munich, Germany) and split into a pump beam with average power of 50 mW and a probe beam with average power of 10 mW. The pump pulses are focused onto a photoconductive THz emitter having a semi-insulating (SI-) GaAs substrate and width of 300 µm between its bias electrodes, which are biased at an alternating-current voltage of 50 V_p-p_. The emitted THz radiation is then collimated, focused through the microfluidic platform, re-collimated and overlapped with the probe beam via a pellicle beamsplitter, and finally focused along with the probe beam through a 0.5 mm thick <110> ZnTe crystal. This is done with the four parabolic mirrors shown in the figure. Such an arrangement has the THz electric field map itself onto the polarization state of the probe beam within the electro-optic crystal [26]. The THz-induced change to the probe polarization is then measured by a quarter waveplate (Thorlabs Inc., Newton, NJ, USA), polarizing beamsplitter (Thorlabs Inc., Newton, NJ, USA), and differential photodetector (Thorlabs Inc., Newton, NJ, USA). For the THz-TDS measurements, the (PET and UHMWPE) microfluidic platforms and thin (PET, PC, PMMA, and PDMS) samples are positioned at the focus of the THz beam, as shown in the figure, while the thick (FEP, PS, HDPE, and UHMWPE) samples are positioned within (and perpendicular to) the collimated THz beam between the first and second parabolic mirrors. The thick samples are positioned in this location to have the beam be well collimated while propagating through the samples. This requirement is not an issue for the microfluidic platforms with thin samples because the depth-of-focus of the focused THz beam is much larger than their thicknesses. The overall system is enclosed by a nitrogen-filled chamber to mitigate the effects of ambient water vapour absorption.

The output of the THz-TDS system is a time-domain waveform that characterizes the electric field of the THz pulse. The waveform is Fourier transformed to extract the THz amplitude distribution for the sample and its reference, being *E*_s_(*f*) and *E*_r_(*f*), respectively, and the THz phase distribution for the sample and its reference, being $\phi_{s}\left(f\right)$ and $\phi_{r}\left(f\right)$, respectively, as a function of the frequency, *f*. The THz amplitude and phase distributions are then used to define the frequency-dependent refractive index of the sample as [27]:

(1) $$n\left(f\right)=1+c\left[\phi_{s}\left(f\right)-\phi_{r}\left(f\right)]/[2\pi fd\right]$$

where *c* is the free-space speed of light and *d* is the sample thickness. The frequency-dependent transmissivity, T(f), and reflectivity, R(f), can then be related to n(f) via:

(2) $$T\left(f\right)=1-R\left(f\right)=1-[n\left(f\right)-1{]}^{2}/[n\left(f\right)+1{]}^{2}$$

The frequency-dependent absorption coefficient, defined in this work as the exponential decay constant for the propagating THz electric field, as opposed to intensity, is then given by:

(3) $$\alpha \left(f\right)=-\ln\left[\frac{1}{T\left(f\right)}E_{s}\left(f\right)/E_{r}\left(f\right)\right]/d.$$

In general, the THz amplitude distributions will be strongest for lower frequencies and decay until the amplitude reaches the noise floor. Thus, the frequency-dependent dynamic range can be defined as the THz amplitude distribution normalized to the noise floor [28], and this can be used to estimate the maximum measurable absorption of the sample. The maximum measurable THz absorption coefficient is [29]:

(4) $$\alpha_{\max}\left(f\right)=\ln\left[\mathrm{DR}\left(f\right)\left(4n/{\left(n+1\right)}^{2}\right)\right]/d,$$

where this $\alpha_{\max}$(*f*) occurs at the frequency for which the THz amplitude distribution attenuates to the noise floor level, for the given sample thickness.

## 3. Results and Discussion

### 3.1. Terahertz Absorption of Potential Polymers for Microfluidic Platforms

The absorption spectra of microfluidic platform materials obtained based on the averaging of five scans of the absorption coefficient spectrum, followed by a moving average low-pass filter to reduce the noise influence in the obtained spectra. 

**Figure 2:**
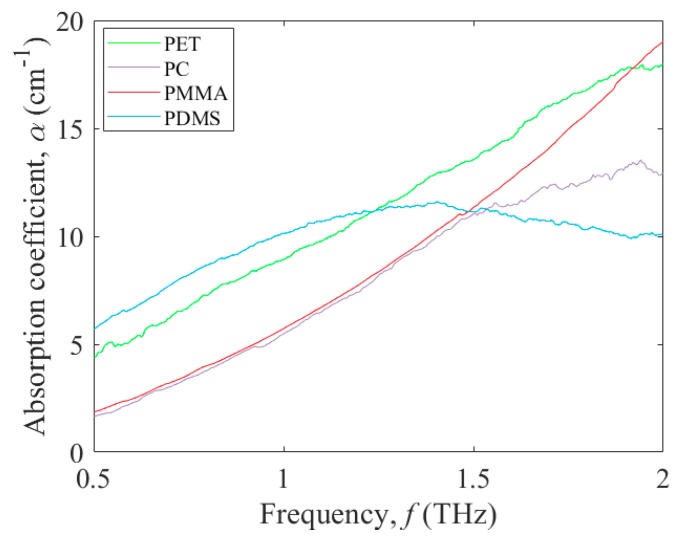
Terahertz absorption coefficient, *α*, versus frequency, *f*, from 0.5 to 2.0 THz for the polar polymers: PET (green curve), PC (purple curve), PMMA (red curve), and PDMS (blue curve).

The characterized polymers are classified into two groups: the selected polar polymers, which include PET, PC, PMMA, and PDMS; and the selected nonpolar polymers, which include FEP, PS, HDPE, and UHMWPE. The selected polar polymers’ THz absorption coefficient, *α*, as a function of frequency, *f*, is shown in Figure 2 for PET (green curve), PC (purple curve), PMMA (red curve), and PDMS (blue curve). For PET, the THz absorption coefficient curve increases from 4.4 cm^−1^ at 0.5 THz to 17.97 cm^−1^ at 2.0 THz. For PC, the THz absorption coefficient curve increases from 1.65 cm^−1^ at 0.5 THz to 12.95 cm^−1^ at 2.0 THz, with a peak of 13.43 cm^−1^ at 1.92 THz. For PMMA, the THz absorption coefficient curve is similar to that of PC in that it increases from 1.85 cm^−1^ at 0.5 THz to 11.04 cm^−1^ at 1.5 THz, but beyond this point it continues to rise up to 19.03 cm^−1^ at 2.0 THz. For PDMS, the THz absorption coefficient values at lower frequencies are the highest of all four polar polymers, but for higher frequencies they become the lowest of all four polar polymers. This manifests as an absorption coefficient curve that increases from 5.7 cm^−1^ at 0.5 THz to a peak of 11.56 cm^−1^ at 1.4 THz followed by a decrease down to 10.06 cm^−1^ at 2.0 THz.

**Figure 3:**
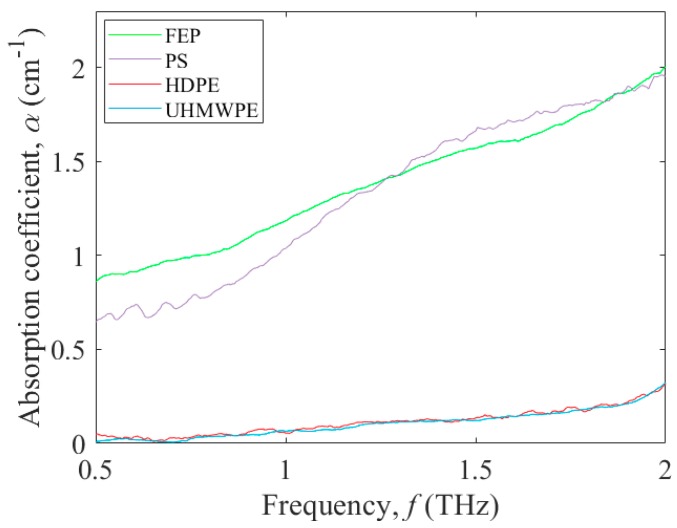
Terahertz absorption coefficient, *α*, versus frequency, *f*, from 0.5 to 2.0 THz for the nonpolar polymers: FEP (green curve), PS (purple curve), HDPE (red curve), and UHMWPE (blue curve).

The THz absorption coefficient, *α*, versus frequency, *f*, of the selected nonpolar polymers is shown in Figure 3 for FEP (green curve), PS (purple curve), HDPE (red curve), and UHMWPE (blue curve). For FEP and PS, the THz absorption coefficient curves increase in a similar manner from 0.86 cm^−1^ and 0.65 cm^−1^ at 0.5 THz, respectively, to 1.95 cm^−1^ at 2.01 THz. For UHMWPE and HDPE, both of which are subsets of polyethylene thermoplastics [30], the THz absorption coefficient curves are also similar, showing an increase from 0.05 cm^−1^ at 0.5 THz to 0.32 cm^−1^ at 2.0 THz. It is clear from these results that the THz absorption coefficients for these nonpolar polymers are far lower than those of the polar polymers. Such an observation makes sense in that the THz radiation more readily couples to the dipole moments of the polar polymers, in comparison to those of the nonpolar polymers, and this is seen as higher absorption [31].

### 3.2. Terahertz Absorption of a Test Fluid within Microfluidic Platforms

To investigate polymers for their ability to function within microfluidic platforms, this study compares microfluidic platforms formed from PET, as a representative high-loss polar polymer, and UHMWPE, as a representative low-loss nonpolar polymer. The comparison is made for these microfluidic platforms using Sylgard^®^ 184 silicone elastomer curing agent (PDMS-CA) as a test fluid. The goal here is to extract the frequency-dependent THz absorption coefficient of the PDMS-CA test fluid with minimal influences from the microfluidic platforms. The PDMS-CA is chosen for this role because it has relatively low values for the THz absorption coefficient, *α*(*f*), spanning roughly 5 cm^−1^ to 10 cm^−1^ and can, thus, clearly distinguish between the capabilities of the microfluidic platforms. The thickness of the test cell, and thus the thickness of the PMMA spacer, is made equal to 2.4 mm to have the thickness lie in the range spanned by 2/*α*(*f*) [24], being 2 to 4 mm. The analysis could also be carried out for contemporary test fluids, such as deionized water and oil, although the microfluidic platform would need to be redesigned to have an appropriate thickness for these fluids’ level of absorption. For the extreme case of high absorption, with deionized water as the test fluid, the microfluidic platforms should be designed with a test cell thickness between 100 to 200 µm. 

**Figure 4:**
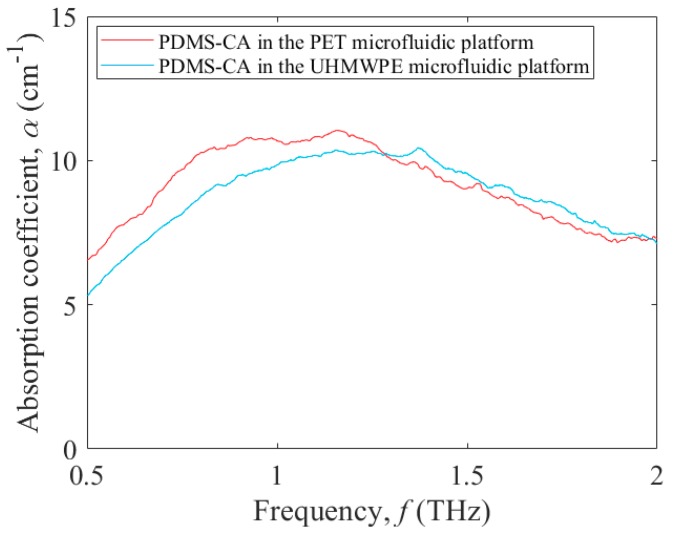
Terahertz absorption coefficient, *α*, versus frequency, *f*, from 0.5 to 2.0 THz for PDMS-CA as the test fluid in PET (red curve) and UHMWPE (blue curve) microfluidic platforms.

Figure 4 shows the THz absorption coefficient, *α*, versus frequency, *f*, of PDMS-CA in the PET microfluidic platform (red curve) and UHMWPE microfluidic platform (blue curve). At lower frequencies, the THz absorption coefficient curve of the PDMS-CA in the PET microfluidic platform is higher than that of the UHMWPE microfluidic platform, while for higher frequencies the two curves are similar. Specifically, the THz absorption coefficients of the PDMS-CA with PET and UHMWPE microfluidic platforms at 0.5 THz are 6.5 cm^−1^ and 5.3 cm^−1^, respectively, while they become equal at 1.3 THz (yielding 10.2 cm^−1^) and again at 2.0 THz (yielding 7.3 cm^−1^). The difference seen between these two microfluidic platforms is a manifestation of their differing dynamic range.

**Figure 5:**
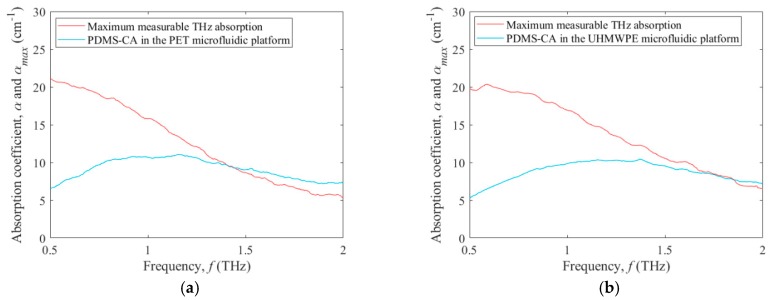
Terahertz absorption coefficient, *α*, and maximum measurable THz absorption coefficient, *α*_max_, versus frequency, *f*, from 0.5 to 2.0 THz for PDMS-CA as the test fluid. The results are shown for the (**a**) PET microfluidic platform and (**b**) UHMWPE microfluidic platform.

The THz absorption coefficients measured within microfluidic platforms will be affected by the dynamic range of the systems—and thus be limited to values below the maximum measurable THz absorption that is defined in Equation (4). This fact, along with observations that THz absorption coefficients typically increase with frequency, suggests that there will be a maximum frequency at which the measured coefficients can be deemed reliable [29]. With these relationships in mind, the THz absorption coefficient for the PDMS-CA test fluid (blue curve) and maximum measurable THz absorption (red curve) are shown in Figure 5a,b versus frequency, *f*, for the PET microfluidic platform and UHMWPE microfluidic platform, respectively. In Figure 5a, the THz absorption coefficient, *α*, curve and maximum measurable THz absorption curve, *α*_max_, suggest that the results for the THz absorption coefficient of the PDMS-CA can only be relied upon for frequencies up to 1.38 THz, because it is at this frequency that *α* ≈ *α*_max_. This contrasts with the results of Figure 5b, which shows the THz absorption coefficient, *α*, curve and maximum measurable THz absorption curve, *α*_max_, intersecting at 1.75 THz. This suggests that the UHMWPE microfluidic platform can yield reliable results for the THz absorption coefficient of the PDMS-CA up to 1.75 THz. The differing bandwidths for the two microfluidic platforms are a manifestation of the differing dynamic ranges of the systems. The higher absorption in the PET microfluidic platform cause it to reach the maximum measurable THz absorption at a lower frequency, in comparison to the UHMWPE microfluidic platform, and this restricts the bandwidth over which its results can be relied upon. This is a fundamental principle that should be considered in designing microfluidic platforms for future THz applications—and interpreting the resulting data.

## 4. Conclusions

In this work, a wide variety of polar and nonpolar polymers were investigated for use in THz spectroscopy within microfluidic platforms. It was found that the THz absorption characteristics vary greatly between the polymers, with the THz absorption coefficients for the nonpolar polymers being far lower than those of the polar polymers. With this in mind, two microfluidic platforms were fabricated: one was based upon PET (with high THz absorption) and the other was based upon UHMWPE (with low THz absorption). It was shown that the differing levels of THz absorption within the polymers yield differing dynamic ranges. This then set differing maximum measurable THz absorption coefficients for the platforms and ultimately limited the bandwidths over which the measured THz absorption coefficients could be relied upon. It was found that the UHMWPE microfluidic platform yielded the lowest THz absorption and thus the highest THz bandwidth. Ultimately, the characterizations and conclusions from such work can play a key role in developing future implementations of THz technology and polymer-based microfluidic systems. 

## Figures and Tables

**Table 1:** Thicknesses (as defined at the centre) of the polymer samples used in the THz-TDS analyses.

Polymer		Thickness (mm)	Preparation
**Polar Polymers**	PET	0.65 ± 0.1	Thermal bonding
	PC	1.00 ± 0.1	Bulk material
	PMMA	1.00 ± 0.1	Bulk material
	PDMS	1.70 ± 0.3	Mixing and curing
**Nonpolar polymers**	FEP	4.30 ± 0.2	Thermal bonding
	PS	4.80 ± 0.2	Thermal bonding
	HDPE	25.4 ± 0.1	Bulk material
	UHMWPE	25.6 ± 0.2	Thermal bonding
